# Neutralizing and non-neutralizing monoclonal antibodies against dengue virus E protein derived from a naturally infected patient

**DOI:** 10.1186/1743-422X-7-28

**Published:** 2010-02-04

**Authors:** John S Schieffelin, Joshua M Costin, Cindo O Nicholson, Nicole M Orgeron, Krystal A Fontaine, Sharon Isern, Scott F Michael, James E Robinson

**Affiliations:** 1 Section of Pediatric Infectious Disease, Department of Pediatrics, Tulane University School of Medicine, New Orleans, LA, USA; 2 Department of Biological Sciences, Florida Gulf Coast University, Fort Myers, FL, USA; 3 Department of Microbiology, University of Washington School of Medicine, Seattle, WA, USA

## Abstract

**Background:**

Antibodies produced in response to infection with any of the four serotypes of dengue virus generally provide homotypic immunity. However, prior infection or circulating maternal antibodies can also mediate a non-protective antibody response that can enhance the course of disease in a subsequent heterotypic infection. Naturally occurring human monoclonal antibodies can help us understand the protective and pathogenic roles of the humoral immune system in dengue virus infection.

**Results:**

Epstein-Barr Virus (EBV) transformation of B cells isolated from the peripheral blood of a human subject with previous dengue infection was performed. B cell cultures were screened by ELISA for antibodies to dengue (DENV) envelope (E) protein. ELISA positive cultures were cloned by limiting dilution. Three IgG1 human monoclonal antibodies (HMAbs) were purified and their binding specificity to E protein was verified by ELISA and biolayer interferometry. Neutralization and enhancement assays were conducted in epithelial and macrophage-like cell lines, respectively. All three HMAbs bound to E from at least two of the four DENV serotypes, one of the HMAbs was neutralizing, and all were able to enhance DENV infection.

**Conclusions:**

HMAbs against DENV can be successfully generated by EBV transformation of B cells from patients at least two years after naturally acquired DENV infections. These antibodies show different patterns of cross-reactivity, neutralizing, and enhancement activity.

## Background

*Dengue viruses *(DENV), members of the genus *Flavivirus*, are the most common cause of mosquito-borne viral diseases in tropical and subtropical regions around the world. Approximately 50 to 100 million people per year are infected with DENV [1]. DENV infections may be asymptomatic, but most often manifest as dengue fever (DF), a self-limited disease. Dengue hemorrhagic fever (DHF) and dengue shock syndrome (DSS) are more severe, life-threatening manifestations of dengue infection. The pathogenesis of DHF/DSS is not completely understood. There are four serotypes of dengue virus (DENV-1, DENV-2, DENV-3, and DENV-4). Infection with one serotype confers lifelong homotypic immunity, but only short term (approximately three to six months) cross protection against heterotypic serotypes [2]. The risk of severe disease is greatest during secondary, heterotypic infections in areas with more than one circulating serotype [3]. There is evidence that prior infection with one type can produce an antibody response that can intensify or enhance the course of disease during a subsequent infection with a different serotype [1,4,5]. The possibility that vaccine components could elicit enhancing antibody responses, as opposed to protective responses, has been a major concern in designing and testing vaccines to protect against dengue infections [6].

The DENV surface contains two proteins: a membrane protein (M) and the envelope glycoprotein (E). E proteins are glycosylated and arranged in homodimers on the viral surface and are involved in receptor binding and entry into susceptible cells [7,8]. The E protein is the primary target for antibody-mediated neutralization and thus the focus of vaccine design. This surface glycoprotein is made up of three domains. The central domain I is flanked on one side by domain II which contains the hydrophobic fusion loop. This loop lies in a pocket between the opposing E protein dimer units and is involved in acid-catalyzed fusion [9]. After virions gain access to an endosome, the lowered pH causes the hinge region of domain I to flex, changing the E protein dimer into a trimer and exposing the fusion loops on domain II. This conformational change at low pH triggers fusion of the viral and cellular endosomic membranes, allowing for nucleocapsid entry into the cytoplasm. Murine monoclonal antibodies (MMAbs) targeting domain I epitopes tend to be non-neutralizing. While there is evidence that some MMAbs binding to domain II epitopes may be neutralizing, others are not [7,10,11]. Domain III, on the opposite side of domain I, contains an immunoglobulin-like structure that is involved in host cell binding [10]. It is also thought to be a major site for serotype-specific antibody-mediated neutralization in mouse models [11-13].

In order to make a safe vaccine, a better understanding of human humoral immune responses to natural DENV infection is required. Although most neutralizing antibodies are directed against the viral envelope protein (E), the precise epitopes that elicit homotypic and heterotypic neutralizing antibodies in naturally infected human subjects have not been characterized and the relationship between neutralizing and enhancing antibodies has not been defined. Studies with monoclonal antibodies provide one approach to identification and characterization of neutralization epitopes. However, to date most anti-dengue monoclonal antibodies are of mouse origin and have been generated from mice immunized with E proteins or live virus [10,14]. The extent to which the human antibody responses elicited by DENV infections target the same or different epitopes is incompletely understood. The purpose of this study was to derive human B cell lines producing human monoclonal antibodies (HMAb) against dengue virus E proteins in order to determine functional properties of antibodies made in response to natural infection in hosts that are actually susceptible to complications of dengue infections. Here we present data demonstrating that it is feasible to isolate dengue virus E protein-specific human B cell lines more than two years after infection.

## Materials and methods

### Viruses and Cells

DENV-1 strain HI-1, DENV-2 strain NG-2, DENV-3 strain H-78, and DENV-4 strain H-42, were obtained from R. Tesh at the World Health Organization Arbovirus Reference Laboratory at the University of Texas at Galveston. Viruses were propagated in the *Macaca mulatta *kidney epithelial cell line, LLC-MK-2, obtained from the ATCC (*Manassas, VA*). LLC-MK-2 cells were grown in Dulbecco's modified eagle medium (DMEM) containing 10% (v/v) fetal bovine serum (FBS) 2 mM Glutamax, 100 U/ml penicillin G, 100 μg/ml streptomycin and 0.25 μg/ml amphotericin B, at 37°C with 5% (v/v) CO_2_. The cells were inoculated with dengue virus stock at 70% to 80% confluency, cultured in DMEM and 10% FBS for seven days, at which time medium was changed to Protein Free Hybridoma Medium (*Gibco, Carlsbad, CA*). After ten days in culture, supernatant fluids were collected and treated with 1% Triton-X 100 to solubilize and inactivate virus. Adherent cells were collected by treatment with trypsin-EDTA for three minutes. Cells were then pelleted by centrifugation at 1000 rpm for 10 minutes. The pellet was re-suspended in 5 ml of PBS containing 1% Triton-X 100. The detergent treated preparations were then mixed thoroughly and aliquoted and frozen at -20°C for later use. K-562 hematopoietic cells (*ATCC, Manassas VA*), used for virus enhancement assays, were grown in RPMI-1640, 10% (v/v) FBS, 2 mM Glutamax, 100 U/ml penicillin G, 100 μg/ml streptomycin and 0.25 μg/ml amphotericin B, at 37°C with 5% (v/v) CO_2_.

### Patient Sample

A patient was identified who had been hospitalized in Singapore with a dengue virus infection in April of 2005. The infection was likely acquired while the patient was traveling in Myanmar. Blood was drawn in September 2007, after informed consent was obtained, and peripheral blood mononuclear cells (PBMCs) were isolated by Ficoll-Hypaque gradient centrifugation and viably frozen in liquid nitrogen. The patient's serum was tested by ELISA and neutralization assays in an attempt to determine the likely infecting serotype. Institutional Review Board approval was obtained for this study at all participating institutions.

### Epstein-Barr Virus Transformation

The production of HMAbs by EBV-transformation of B cells has been described elsewhere [15-17]. Briefly, viably cryopreserved PBMCs were thawed, washed in Hank's Buffered Salt Solution, and inoculated with EBV (B95-8 strain). Cells were suspended in RPMI containing 20% FBS, Primacin^® ^(*InVivoGen, San Diego, CA*) and 2 g/ml CpG 2006 (*Midland Certified Reagent Co., Midland, TX*) and plated at 10^4 ^cells per well in 96 well tissue culture plates previously seeded with approximately 50,000 irradiated mature macrophages per well derived from PBMC of healthy blood donors which served as "feeder cultures" that promote outgrowth of transformed B cells. Antibody-positive wells that contained growing cells were sub-cultured at several dilutions and re-screened by ELISA. Cell lines that continued to grow and produce antibody during several low cell density passages were finally cloned at limiting dilution. Definitively cloned cell lines were expanded to grow as suspension cultures in stationary 490 cm^2 ^roller bottle cultures (*Corning, Corning, NY*) from which cell culture fluid was harvested weekly. HMAbs were purified from one to two liters of culture supernatant by Protein A affinity chromatography. The IgG subclass and light chain type of each antibody was determined by reactivity with MMAbs to the four heavy chain subclasses (*Southern Biotech, Birmingham, AL*) and polyclonal goat antibodies to kappa and lambda chains by ELISA using established methods.

### ELISA to Detect Human and Murine Anti-Dengue Virus Monoclonal Antibodies

Transformed B cell cultures were screened for antibody production using a modification of an immunoassay described previously in which virus envelope glycoproteins are immobilized in wells coated with Concanavalin A (ConA) a plant lectin that binds carbohydrate moieties on glycoproteins of a variety of enveloped viruses [16]. 96-well plates (*Costar*^®^, *Corning, Corning, NY*) were coated with ConA (*Vector Laboratories, Burlingame, CA*) at 25 ug/ml in 0.01 M HEPES (*Gibco, Carlsbad, CA*) and 100 l per well for one hour. The wells were washed and solubilized DENV was incubated for one hour. A requirement of this assay is that virus must be grown in serum free medium so that viral glycoproteins can be captured in ConA coated wells. Media containing FBS has glycoproteins that will bind to ConA and block capture of DENV E protein resulting in low OD readings. After a wash step with PBS containing 0.1% (v/v) Triton-X 100, un-reacted ConA binding sites in the wells were blocked with RPMI Medium 1640 and 10% FBS for 30 minutes. Culture fluids from each 96 well culture plate containing EBV transformed B cells were transferred to corresponding wells of assay plates coated with dengue E proteins and incubated for one hour at room temperature. Undiluted supernatant of murine MAb 3H5 (ATCC HB-46), which binds to DENV-2, was used as a positive control during the screening process [18]. Negative controls consisted of LLC-MK-2 culture fluid grown in parallel with no virus as well as vaccinia expressed HIV envelope glycoprotein's from the ADA strain of HIV-1 produced in serum free medium. The wells were again washed and then incubated with 100 ul of peroxidase-conjugated goat anti-human IgG-gamma (*Zymed, San Francisco, CA*) or peroxidase-conjugated affinity purified anti-mouse IgG (*Rockland, Gilbertsville, PA*) diluted 1:2000 in PBS-0.5% (v/v) Tween 10% (w/v) whey (*BiPro, Le Sueur, MN*) and 10% (v/v) FBS for one hour. After a final wash step, color was developed with 100 μl/well tetramethylbenzidine-peroxide (TMB)-H_2_O_2 _as substrate for peroxidase. The reaction was stopped after 4 minutes by adding 1% phosphoric acid and color was read as optical density (OD) at 450 nm. All steps in this ELISA were performed at room temperature.

### Biolayer Interferometry Binding Assays

Real time binding assays between purified antibodies and purified DENV E proteins were performed using biolayer interferometry with an Octet QK system (*Fortebio, Menlo Park, CA*). This system measures light interference on the surface of a fiber optic sensor, which is directly proportional to the thickness of molecules bound to the surface. Targets of interest are chemically tethered to the surface of the sensor using biotin-streptavidin interactions. Binding of a partner molecule to the tethered target results in thickening of the surface, which is monitored in real time. Purified, recombinant, 80% truncated DENV 1-4 E proteins were obtained from Hawaii Biotechnology (*Aiea, HI*) [19,20]. E proteins were biotinylated for 30 minutes at room temperature using a 5:1 molar ratio of NHS-LC-LC-Biotin (*Pierce/ThermoFisher, Rockford, IL*) and dialyzed against PBS to remove unreacted biotinylation reagent. Biotinylated E proteins were coupled to kinetics grade streptavidin high binding biosensors (*Fortebio, Menlo Park, CA*) at several different concentrations. E protein binding concentrations that gave a signal between 0.8 and 1.2 nm binding to the sensor surfaces within 200 seconds were used for antibody binding studies. Unbound E proteins were removed from the surface of the sensors by incubation in PBS. Probes coupled to E protein were allowed to bind to antibodies at several different concentrations, and binding kinetics were calculated using the Octet QK software package, which fit the observed binding curves to a 1:1 binding model to calculate the association rate constants. Antibodies were allowed to dissociate by incubation of the sensors in PBS. Dissociation kinetics were calculated using the Octet QK software package, which fit the observed dissociation curves to a 1:1 model to calculate the dissociation rate constants. Association and dissociation rate constants were calculated using at least two different concentrations of antibody. Equilibrium dissociation constants were calculated as the kinetic dissociation rate constant divided by the kinetic association rate constant.

### Antibody Cross-competition assay

To determine whether HMAbs recognized overlapping or non-overlapping sites, we tested the MAbs for cross-competition with each other and with MMAb 4G2 using an adaptation of our previously described method [21,22]. Detergent solubilized dengue E protein in serum free culture fluid was immobilized in Con A coated wells at room temperature. The plates were washed and blocked for 30 minutes at room temperature. Purified HMAbs, MAb 4G2 or dilution buffer was incubated in the wells for 30 minutes at room temperature. Biotinylated HMAbs were then added to the wells at dilutions that gave less than maximal binding and incubated for one hour at room temperature. Bound biotinylated HMAb was detected with horseradish peroxidase streptavidin (*Vector, Burlingame, CA*). After the wells were washed, the ELISA was completed as described above.

### Focus-forming-unit Reduction Neutralization Titer

LLC-MK-2 target cells were seeded at a density of approximately 500,000 cells in each well of a 12-well plate 24 h prior to virus inoculation. Approximately 100 focus forming units (ffu) of virus were incubated with heat inactivated patient serum or purified HMAb in serum-free DMEM for one hour at room temperature. Virus mixtures were allowed to infect confluent target cell monolayers for one hour at 37°C, with rocking every 15 minutes, after which time the inoculum was aspirated and overlaid with fresh MEM/10% (v/v) FBS containing 1.2% (w/v) microcrystaline cellulose (*Avicel, FMC, Newark, DE*). Infected cells were then incubated at 37°C with 5% CO_2 _for two days (DENV-4), three days (DENV-1 and -3), or four days (DENV-2). Infected cultures were fixed with 10% (w/v) formalin overnight at 4°C, permeabilized with 70% (v/v) ethanol for 20 minutes, and rinsed with PBS prior to immunostaining. Virus foci were detected using specific mouse anti-DENV E protein MAb E60 (obtained from M. Diamond at Washington University), followed by horseradish peroxidase-conjugated goat anti-mouse immunoglobulin (*Pierce, Rockford, IL*), and developed using AEC chromogen substrate (*Dako, Carpinteria, CA*). Results are expressed as pooled data from two independent experiments with three replicates each.

### Enhancement Assay

Enhancement assays were conducted using DENV-1 in K-562 hematopoietic cells. Varying concentrations of each HMAb were incubated with 7,500 ffu of virus for 1 hour at 37°C in 200 μl of serum free RPMI-1640, then added to 75,000 cells in 300 μl of complete medium in a 24-well plate and incubated at 37°C with 5% CO_2 _for 3 days. RNA was extracted from cell lysates using the RNeasy Mini kit (*Qiagen, Valencia CA*). Quantitative RT-PCR was performed with a DENV-1 specific primer pair (D1S and D1C) that produces a 490 bp product from the NS1 region, using a LightCycler 480 II (*Roche, Indianapolis, IN*) and a one step LightCycler RNA Master SYBR Green I kit (*Roche*) [23]. Amplification conditions were 61°C for 30 min, 95°C for 30 sec, and 45 cycles of 95°C for 5 sec, 61°C for 20 sec, and 72°C for 30 sec.

## Results

### ELISA to Screen for IgG Antibody Production

**Figure 1:**
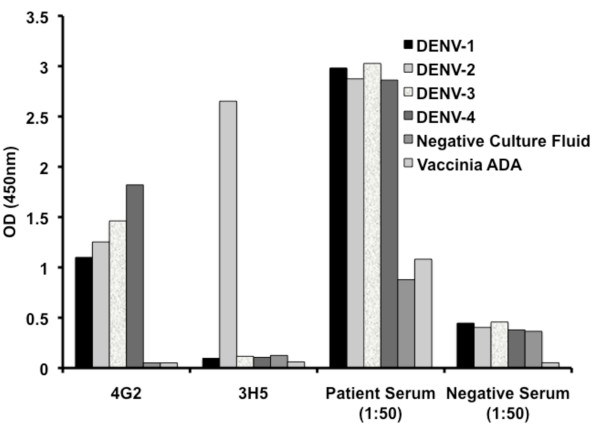
**Validation of the ConA ELISA**. DENV E protein from serotypes 1-4 derived from DENV infected LLC-MK-2 cells was immobilized by ConA. Reactivity with patient serum and dengue negative human serum diluted in PBS/Tween as well as MMAbs 4G2 and 3H5 was measured by detection with HRP anti-human and anti-mouse IgG, respectively. MMAb 4G2 were tested at 5 ug/ml. Mouse MAb 3H5 was tested as undiluted culture fluid. Negative controls of uninfected LLC-MK-2 culture fluid and Vaccinia ADA were also tested.

We first determined that DENV E proteins captured in assay wells coated with ConA and still retained antigenicity. The ELISA data presented in Figure 1 shows that antibodies in a dengue positive serum reacted strongly with ConA immobilized E proteins of all four serotypes while a dengue negative human serum showed only low levels of background reactivity (Figure 1). Equivalent low background reactivity (O.D. <0.300) was also seen when the dengue positive and negative sera were tested in ConA coated wells incubated with tissue culture fluid lacking dengue E proteins. As further controls, we also tested two murine MAbs with known binding to dengue E proteins, 3H5 and 4G2 (ATCC HB-46 and HB-112, respectively), for reactivity in this assay. 3H5 is quite strain restricted to DENV-2 while 4G2 cross-reacts with all dengue serotypes and other flaviviruses as well. In the Con A assay these MAbs reacted as expected: 4G2 bound the four serotypes and 3H5 bound only to Dengue 2. [18].

### Isolation of B Cell Lines Producing DENV Specific HMAbs

We identified a patient with a history of DENV infection approximately two years previously. Serum from this patient contained IgG antibodies that cross-reacted with E proteins of all four strains of DENV by ELISA (Figure 1). B cells were transformed and screened for IgG antibodies binding to DENV-2 E proteins immobilized in ConA coated wells of assay plates. IgG antibodies reacting with DENV-2 E protein were detected in 11 of 558 (2%) of the EBV transformed B cell cultures from the patient sample. From three of the initially positive cultures we established cloned B cell lines that stably produced three MAbs, designated 2.3D, 3.6D and 4.8A. The IgG subclass and light chain type of each antibody was determined. Both 3.6D and 4.8A were IgG1 with kappa light chains while 2.3D was IgG1 with lambda light chains.

**Figure 2:**
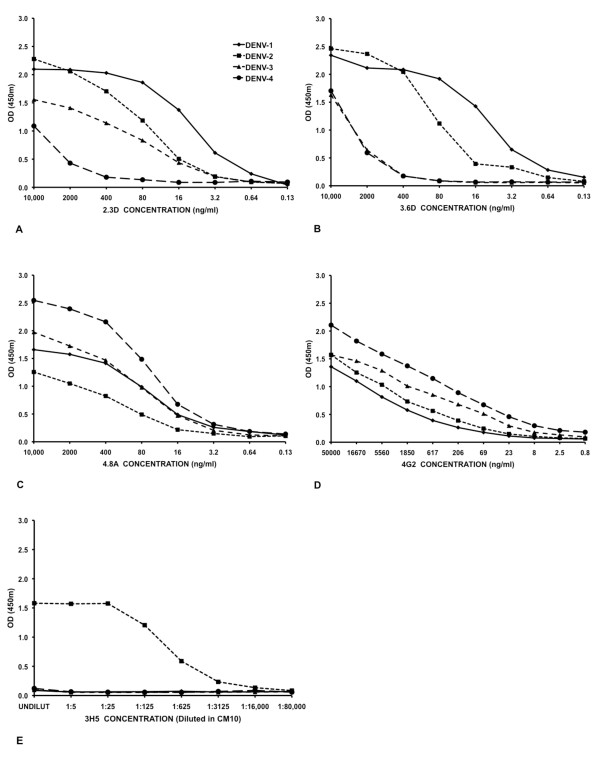
**Serial five fold dilutions of each HMAb**. Starting at 10,000 ng/ml, Human and Mouse MAbs were tested by ConA ELISA against each dengue virus serotype. A HMAb 2.3D. B 3.6D. C 4.8A. D. 4G2 (dilutions were started at 50,000 ng/ml). E. 3H5 (tested as dilutions of culture fluid).

Next we titered the binding activities of protein A purified HMAbs with ConA-immobilized E proteins from each DENV serotype (Figure 2). MMAbs 3H5 and 4G2 served as positive controls. Each MAb bound to E proteins in a dose dependent fashion (Figure 2). There was no reactivity with negative controls consisting of LLC-MK-2 culture fluid grown in parallel with no virus (data not shown). The patterns of cross-reactivity differed for the HMAbs; HMAb 2.3D bound strongly to DENV-1, -2, moderately to DENV-3. Reactivity of 2.3D with DENV-4 was observed only at high concentrations (10 μg/ml) but binding activity dropped off rapidly with antibody dilution. HMAb 3.6D bound strongly to DENV-1 and -2, but binding to DENV-3 and -4 only occurred at high concentrations (≥2 μg/ml). HMAb 4.8A bound strongly to DENV-1, -2, and -3, and moderately well to DENV-4. As expected, the control mouse MMAb 3H5 bound only to DENV-2 while the highly cross-reactive MMAb 4G2, bound to all four serotypes (Figures 1, 2D and 2E).

### Cross-competition between HMAbs

**Figure 3:**
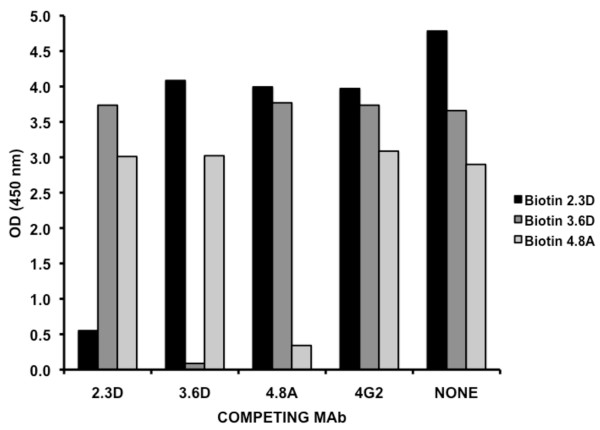
**Cross-competition between HMAbs**. A cross-competition assay was performed to determine whether the three HMAbs and MMAb 4G2 recognized overlapping or non-overlapping sties on DENV-1 E protein. Purified MMAb 4G2 and HMAbs were incubated with biotinylated HMAbs, washed, and the presence of biotinylated antibodies was detected using streptavidin.

A cross-competition assay was performed to determine whether the three HMAbs recognized overlapping or non-overlapping sties on DENV-1 E protein. We tested the ability of each HMAb to block binding of each biotin labeled HMAb. As shown in Figure 3, each HMAb was able to block itself (e.g. 4.8A blocked binding of biotin 4.8A) but was unable to block the other two HMAbs. These results indicate that the three HMAbs recognize non-overlapping sites on DENV E proteins. In addition preliminary results indicated that MMAb 4G2 did not block binding of any of the HMAbs. Taken together our results demonstrate the three human MAbs recognize distinct non-overlapping sites, which are also independent of the 4G2 epitope.

### Neutralization

**Figure 4:**
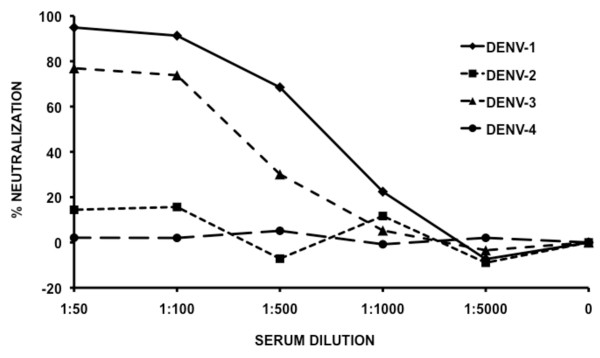
**Virus neutralization by patient serum**. Approximately 100 FFU of virus were incubated with serial dilutions of heat inactivated patient serum and then allowed to infect LLC-MK-2 cells. Following an incubation period, virus foci were detected using specific mouse anti-DENV E protein MMAb E60.

To determine which serotype was likely to have infected the patient we performed serum neutralization assays against each of the four strains of DENV (Figure 4). The patient serum showed little or no neutralization activity against either DENV-2 or DENV-4. The highest level of neutralization activity was seen against DENV-1, suggesting that this may have been the original infecting serotype. In support of this, published data from Myanmar suggests that beginning in 2001, DENV-1 was the predominant circulating strain [24]. The serum also showed considerable neutralization activity against DENV-3, however since the patient described only a single dengue-like illness event, the ability of the patient's serum to neutralize DENV-1 and DENV-3 most likely reflects the development of cross-reactive neutralizing antibodies rather than exposure to a second serotype. Because no tests were initially performed to determine the infecting serotype, it is impossible to know for certain which one it was.

**Figure 5:**
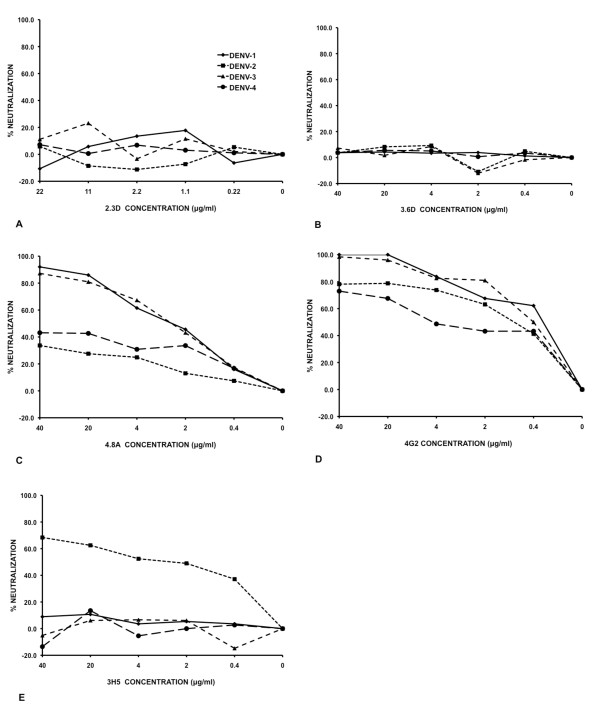
**Virus neutralization by HMAbs**. Approximately 100 FFU of virus were incubated with serial dilutions of purified monoclonal antibody. Virus mixtures were allowed to infect LLC-MK-2 cells and then incubated at 37C. Virus foci were detected using anti-DENV E protein MMAb E60. Results are expressed as pooled data from two independent experiments with three replicates each. A HMAb 2.3D. B 3.6D. C 4.8A. D 4G2. E 3H5.

We tested the neutralizing activity of the three HMAbs against representative strains of all four DENV serotypes (Figure 5). Neither the 2.3D nor 3.6D antibodies showed neutralizing activity against any DENV serotype at any concentration tested. In contrast, the 4.8A antibody was showed potent neutralizing activity against both DENV-1 and -3, with fifty percent neutralization at approximately 3 g/ml. Although HMAb 4.8A also showed some weak inhibitory activity against DENV strains 2 and 4, the level of inhibition did not reach 50% neutralization activity and thus did not meet the criteria for neutralizing activity.

### Enhancement

Both human polyclonal serum and mouse monoclonal antibodies have been shown to enhance dengue virus infections in F_c _receptor bearing cells that otherwise exhibit low susceptibility to DENV infection [25-27]. To determine if the HMAbs show *in vitro *antibody dependent enhancement (ADE) properties, we infected human K562 hematopoietic cells with DENV-1 in the presence of increasing concentrations of the antibodies. We used the K562 cell line because it expresses only the F_c _gamma RII (CD32) receptor and thus provides a simple and well-characterized system for the study of ADE [28]. DENV-1 was used since all three of the human monoclonal antibodies bound well to E protein of this serotype (Figures 1 and 2), and the 4.8A antibody was highly neutralizing against DENV-1 (Figure 5).

**Figure 6:**
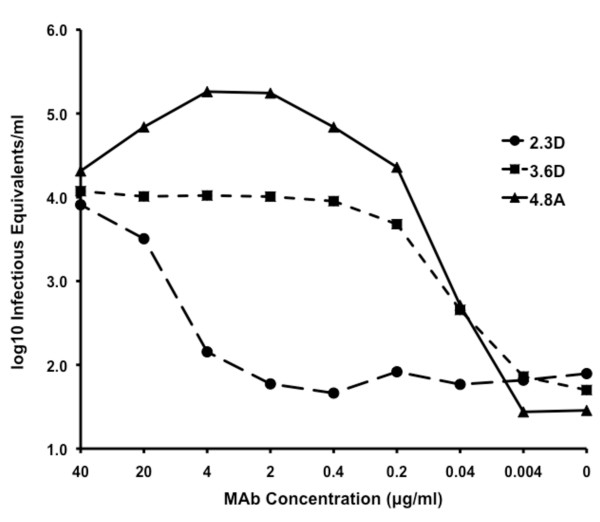
**Enhanced DENV uptake by K562 cells in the presence of HMAbs**. Human K562 cells were infected with DENV-1 in the presence of increasing concentrations of the antibodies. RNA was extracted from cell lysates and quantitative RT-PCR was performed with a DENV-1 specific primer pair specific for the NS1 region as a measure of infectious equivalents. A HMAb 2.3D. B 3.6D. C 4.8A.

The results, shown in Figure 6, indicate that the all three HMAbs were able to enhance viral infection, but they did so with different patterns. HMAbs 3.6D and 4.8A enhanced infection at relatively low concentrations (0.04 g/ml) and the amount of enhancement rose with increasing HMAb concentrations. Enhancement induced by the non-neutralizing 3.6D HMAb reached a plateau above 0.4 g/ml, while enhancement induced by the 4.8A HMAb peaked and subsequently fell at higher concentrations, consistent with its observed neutralization activity. The 2.3D HMAb only showed evidence of enhancement at concentrations above 4 g/ml, consistent with the lower affinity this HMAb has for the DENV-1 E protein (see below). Interestingly, we also observed that the three HMAbs differed markedly in their ability to enhance dengue infection in vitro\with the neutralizing HMAb 4.8A showing the greatest effect.

### Quantitation of HMAb/E protein binding affinity

**Figure 7:**
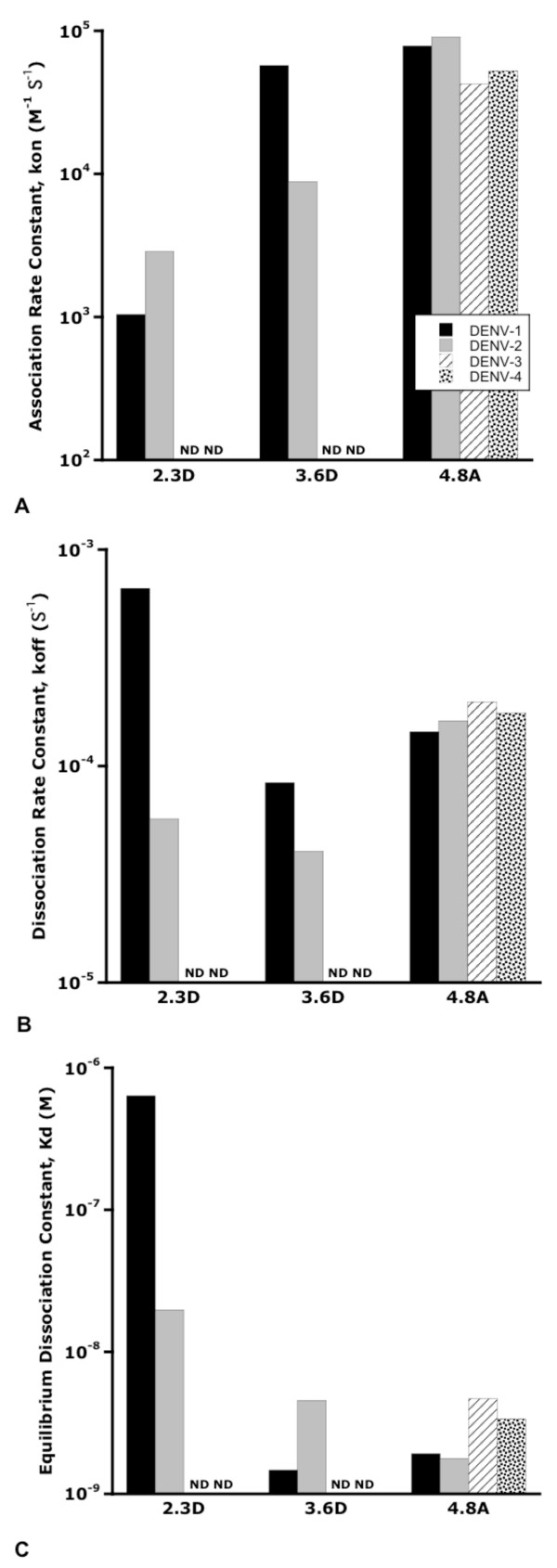
**HMAb measured by biolayer interferometry**. Label free, real time binding analysis between the HMAbs 2.3D, 3.6D and 4.8A and purified, recombinant E protein from each DENV serotype to measure binding affinity was performed. The affinities of 2.3D and 3.6D for DENV 3 or 4 E proteins could not be measured due to Ab aggregation at the higher concentrations needed to detect lower affinity binding. A: Association rate constants (kon) for Ab:E protein interactions. B: Dissociation rate constants (koff) for Ab:E protein interactions. C: Equilibrium dissociation constants (Kd) for HMAb:E protein complexes.

To confirm the HMAb specificity for DENV E proteins and to quantitate the affinity of each antibody for the different DENV strains, we used biolayer interferometry to examine binding between the antibodies and purified, recombinant E protein from each DENV serotype. In these experiments, the E proteins were chemically coupled to biotin and conjugated to the surface of streptavidin-coated fiber optic probes. The conjugated probes were placed in solutions with different concentrations of each antibody. Binding of the antibodies to the E proteins on the surface of the probes was measured by the change in interference from light reflected from the surface of the probe. After binding, the probes were placed in a solution without any antibody to similarly measure the antibody/E protein dissociation. Kinetics of on (Figure 7A) and off (Figure 7B) rates and equilibrium dissociation constants (Figure 7C) were calculated assuming a 1:1 binding ratio using the manufacturer's software (Figure 7). As expected from the patient serum neutralization results and the HMAb ELISA results, all three of the antibodies bound to DENV-1 as well as DENV-2 E protein. HMAb 2.3D showed the weakest binding, with dissociation constants of 2 × 10^-8 ^M for DENV-2 and 6 × 10^-7 ^M for DENV-1. The affinity of HMAb 3.6D was somewhat higher, with dissociation constants of 2 × 10^-9 ^M for DENV-1 E and 5 × 10^-9 ^M for DENV-2 E protein. The increased affinities seen with the 3.6D antibody were due to both increased binding (on rate) kinetics, as well as decreased dissociation (off rate) kinetics. The low binding activities of 2.3D and 3.6D against DENV-3 or -4 E proteins precluded measurement of affinities of these antibodies. HMAb 4.8A showed high affinity binding to all four DENV E proteins with dissociation constants in the 2-5 × 10^-9 ^M range. Binding was slightly better with the DENV-1 and -2 E proteins (2 × 10^-9 ^M) than with the DENV-3 and -4 E proteins (3-5 × 10^-9 ^M).

The broad binding reactivity of MAb 4.8A against the 4 serotypes of DENV contrasts sharply with the DENV-1 and -3 specificity observed in the neutralization assays with this antibody. The ConA ELISA and biolayer interferometry binding assays do not reproduce the subtleties of binding to the surface of an assembled virion. While the purified E proteins are apparently correctly glycosylated, they are truncated at the pre-anchor domain. Therefore, they are not interacting with a lipid membrane and do not form complexes with neighboring E subunits as on the surface of an infectious virion. While 4.8A exhibits potent neutralizing activity against DENV-1 and -3, its target epitope may be sufficiently shielded or altered on DENV-2 and -4 viral particle E proteins to lower this neutralization activity.

## Discussion

In this study we have demonstrated that it is feasible to derive human B cell lines producing HMAbs specific for dengue virus E proteins. The three IgG HMAbs reported here were produced by EBV transformation of circulating memory B cells obtained from a patient who had dengue fever at least two years before. One HMAb, 4.8A, was broadly cross-reactive by ELISA with all four dengue serotypes. HMAb 2.3D bound to DENV-1, 2, 3 by ELISA, while 3.6D reacted with only DENV-1 and 2 E proteins by ELISA. Cross-competition binding assays performed with DENV-1 E proteins indicate that the three HMAbs recognize distinct sites. Of the three HMAbs only 4.8A showed potent neutralizing activity against DENV-1 and DENV-3 and little or no inhibitory activity against DENV 2 and 4. The neutralizing activity of 4.8A mirrored closely that found in the patient's serum. It is not clear why 4.8A showed lower neutralization activity against DENV-2 and 4 even though it reacted well to these serotypes in ELISA and biolayer interferometry assays using disrupted or monomeric E protein. Very likely there are subtle differences of epitope exposure on viral particles in the different serotypes. Neither of the two other HMAbs, 2.3D and 3.6D, was able to neutralize DENV.

All three HMAbs demonstrated concentration dependent enhancement of infection when antibody was incubated with virus prior to infecting F_c _receptor-bearing cells. Antibody Dependent Enhancement (ADE) was first proposed by Hawkes in 1964 who theorized that pre-existing antibody, either neutralizing but at sub-neutralizing concentrations or non-neutralizing, binds to the viral particle and enhances the efficiency of viral uptake into the target cell [29]. Halstead described this *in vitro *phenomenon in DENV in 1970 [4]. Antibody dependent enhancement characteristics have been found with both neutralizing and non-neutralizing anti-DENV MMAbs [5]. The non-neutralizing anti-E protein Ab described by Huang et al demonstrated a positive correlation between enhancement and antibody concentration similar to that seen with HMAbs 2.3D and 3.6D. Our neutralizing HMAb 4.8A also showed a drop in enhancement activity at higher concentrations, consistent with its presumed ability to block viral entry at full Ab occupancy. Enhancement of infection by HMAbs correlates well with affinity. 3.6D and 4.8A bind tightly to DENV-1 E (K_d _= 10^-9 ^M) and they enhance at low concentrations, while 2.3D, which binds less tightly (K_d _= 10^-7 ^M), enhances only at higher concentrations. We also noted that our three HMAbs showed different levels of enhancement that were not explained by affinity. Curiously, the only neutralizing HMAb, 4.8A, showed the greatest enhancement. Although HMAb 4.8A appears to neutralize and enhance in the same range of concentrations (see Figures 5C and 6), each characteristic was measured *in vitro *using a different assay system with different concentrations of virus. We do not know if this will be a consistent phenomenon associated with neutralizing HMAbs. In addition, the relationship between ADE and neutralizing versus non-neutralizing antibodies needs to be more fully explored in cells with different types of F_c _receptors.

Current information on mapping of neutralization epitopes of dengue viruses is based almost entirely on studies with MMAbs that were generated by short-term immunization of mice with infectious virus particles or with purified viral proteins [10,14]. A major drawback to studies with murine antibodies is that dengue virus infection does not occur naturally in mice and significant disease is usually only achieved through intracerebral inoculation or the use of genetically modified mice with immune deficits. The mouse is not a model for DHF or DSS. Human and mouse antibody repertoires are also distinctly different [30]. The variable regions of the heavy chain (V_H_) and lambda light chains (V) have a significantly greater number of combinations in humans than in mice. The germ line complexity of the D_H _and J_H _loci is also greater in humans and the greater length of the CDR-H3 region of the heavy chain allows for more complex binding surfaces. Length and amino acid utilization is very different in this region in humans compared to mice. In humans, this region is able to form grooves, cavities and knobs, increasing the potential range of epitope recognition [31,32].

In addition, humans and mice differ in their major histocompatability complex class II and I gene regions and therefore will present similar antigens differently. Accordingly, human and mouse repertoires may be more, or less, likely to target certain epitopes or they may target similar epitopes but recognize different conformations on them [33,34]. We have incomplete knowledge of how mouse and human antibody responses to dengue viruses differ. However if antibody plays any role in the pathogenesis of DHF/DSS, it is obviously important to focus studies of antibody responses in the host species in which DHF/DSS occurs.

## Conclusions

HMAbs specific for DENV E proteins can be generated by EBV transformation of B cells from patients at least two years after naturally acquired dengue infections. We have generated three such antibodies that recognize three distinct antigenic sites, exhibit varying degrees of serotypic cross-reactive, and show differences in neutralizing, non-neutralizing and enhancing activity. Our results show that it will be possible to generate libraries of HMAbs that will allow a more complete understanding of the role antibodies play in protection and pathogenesis of DENV infections.

## Competing interests

The authors declare that they have no competing interests.

## Authors' contributions

JSS participated in the study design and coordination, performed the EBV transformation, the HMAb purification, and ELISA titrations, analyzed the data and drafted the manuscript. JMC participated in the study design and coordination, performed the neutralization assays, analyzed the data and drafted the manuscript. CON performed the enhancement assays and helped perform the neutralization assays. NMO performed the biotinylation of HMAbs and competition assays. KAF performed the biolayer interference affinity experiments. SI, SFM, and JER conceived and designed the project, coordinated the study, analyzed the data, and helped draft the manuscript. All authors read and approved the final manuscript.
